# Transcriptional Profiling of Serogroup B *Neisseria meningitidis* Growing in Human Blood: An Approach to Vaccine Antigen Discovery

**DOI:** 10.1371/journal.pone.0039718

**Published:** 2012-06-22

**Authors:** Asa K. Hedman, Ming-Shi Li, Paul R. Langford, J. Simon Kroll

**Affiliations:** Section of Paediatrics, Department of Medicine, Imperial College London, St. Mary’s Campus, London, United Kingdom; Health Protection Agency, United Kingdom

## Abstract

*Neisseria meningitidis* is a nasopharyngeal commensal of humans which occasionally invades the blood to cause septicaemia. The transcriptome of *N. meningitidis* strain MC58 grown in human blood for up to 4 hours was determined and around 10% of the genome was found to be differentially regulated. The *nuo*, *pet* and *atp* operons, involved in energy metabolism, were up-regulated, while many house-keeping genes were down-regulated. Genes encoding protein chaperones and proteases, involved in the stress response; complement resistant genes encoding enzymes for LOS sialylation and biosynthesis; and *fHbp* (NMB1870) and *nspA* (NMB0663), encoding vaccine candidates, were all up-regulated. Genes for glutamate uptake and metabolism, and biosynthesis of purine and pyrimidine were also up-regulated. Blood grown meningococci are under stress and undergo a metabolic adaptation and energy conservation strategy. The localisation of four putative outer membrane proteins encoded by genes found to be up-regulated in blood was assessed by FACS using polyclonal mouse antisera, and one (NMB0390) showed evidence of surface expression, supporting its vaccine candidacy.

## Introduction

The Gram-negative bacterium *Neisseria meningitidis* is a human commensal colonizing the nasopharynx, which occasionally invades the bloodstream of susceptible individuals (mainly infants and young people) to cause diseases with substantial morbidity and mortality – septicaemia and meningitis [1], [2]. In the course of invasive infection, meningococci evade innate immune responses – in particular the complement system – and survive in the face of limited availability of micronutrients, such as iron, that are sequestered by the host. Survival in the hostile environment of blood requires the meningococcus to differentially regulate the expression of its genes [3]–[6].

Animals such as the mouse and rat have been widely used as convenient models to investigate which meningococcal genes contribute to bacterial survival in blood, and so to virulence. For example, 73 meningococcal genes that contribute to survival in the blood have been identified using an infant rat model [7]. However, major differences between the nature of meningococcal infection in such animals and in man mean that there must be important reservations in interpreting such studies. To address these, “bridging studies” using human plasma, serum and blood have been undertaken: for example, the serum bactericidal assay is the mainstay for vaccine testing [8]. A whole human blood *ex vivo* meningococcal infection model has been developed to investigate vaccine-induced serum bactericidal activity and the contribution that blood cells play in controlling infection [9], [10]. A single study has evaluated the transcriptome of meningococci grown in human serum [11], and one further study, published during the preparation of this manuscript, has investigated the transcriptome in blood-grown organisms [12].

Like the authors of this last-cited work, we have sought to determine the transcriptome of *N. meningitidis* when grown in non-bactericidal whole human blood (the natural condition of meningococcal septicaemia in infants), with a view to identifying genes that may be important for bacterial survival and those encoding potential vaccine candidates, identified by being upregulated during bacteraemia and exposed on the bacterial surface. Our results are broadly in agreement with those of Enrique *et al*. [12]. We have delineated aspects of the bacterial stress response induced by whole blood, demonstrated up-regulation of genes encoding current key candidate vaccine antigens –fHbp (factor H binding protein) and NHBP (neisserial heparin binding antigen), and identified further candidates with comparable desirable properties. Among these, we have identified NMB0390 in particular as a sequence-conserved and potentially surface exposed protein, expressed in the course of this model of bloodstream infection, that we consider merits further study as a potential vaccine candidate.

## Results

### Characterization of a whole human blood model of meningococcal infection for transcriptional studies

We sought to establish a meningococcus-human blood co-cultivation model, in which *N. meningitidis* grows in fresh human blood at an infection level comparable to that found in patients with meningococcal sepsis [13], from which we could recover sufficient bacterial RNA for transcriptome analysis without amplification. Two healthy adults were identified whose blood had no bactericidal activity against *N. meningitidis* serogroup B strain MC58 – so called non-killers [14].

**Figure 1:**
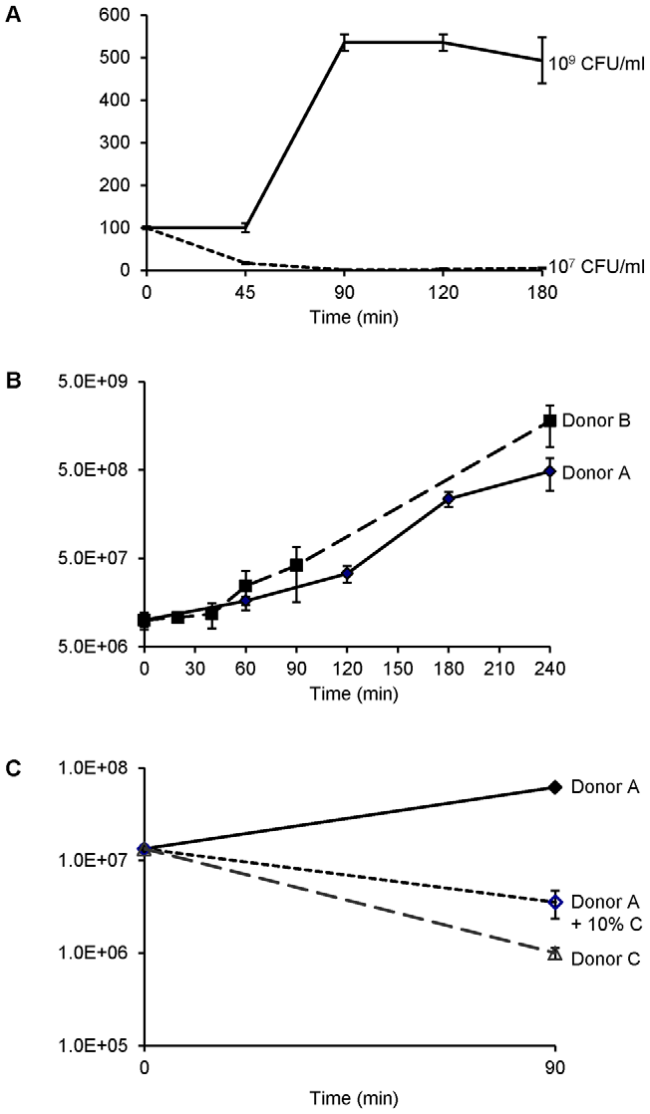
Growth of meningococci (strain MC58) in whole human blood. Meningococcal growth is shown by CFU obtained at time points after initial inoculation. Error bars indicate SEM of biological replicates. (**A**) Growth of meningococci in donor C blood expressed as percent of initial inoculum, 10^7^ (dashed line) or 10^9^ (solid line) CFU per ml of blood. (**B**) Growth of meningococci in donor A (solid line) and B (dashed line) blood. (**C**) Growth of meningococci in donor A (solid line) and C (dashed line) blood, and donor A blood “doped” with 10% of donor C plasma (dotted line).

We began by determining an infection load, representative of bacterial numbers found in septicaemic patients, that would yield sufficient RNA (5 µg) for direct labelling without prior amplification, a step known to introduce transcriptome bias [15]. Employing real-time PCR technology Ovstebo *et al*. [13] found an average of 10^7^ CFU (range 10^5^–10^9^) meningococci per ml of blood from paediatric patients with septic shock. Exploring within this range, in experiments using bactericidal and non-bactericidal fresh whole blood, we found that using 10^5^–10^6^ CFU per ml inoculum we were not able to obtain a sufficient amount of bacterial RNA for direct labelling. Conversely, on using an inoculum of 10^9^ CFU per ml, although sufficient amounts of RNA were obtained, meningococci grew in a donor’s blood that was otherwise bactericidal when using 10^7^ per ml inoculum (Figure 1A). This suggests that normally adequate meningococcal killing mechanisms in this donor’s blood were overwhelmed by the presence of high numbers of bacteria. However, using 10^7^ per ml inoculum resulted in meningococcal numbers increasing steadily over the 240 minute time course (Figure 1B) in contrast to the finding in experiments in which non-killer’s blood was doped with 10% killer’s plasma (Figure 1C). Using 10^7^ per ml inoculum, 5 µg or more of meningococcal RNA could be obtained from aliquots harvested at each time point (0, 20, 40, 60, 90 and 240 min), with very low levels of human RNA contamination (Figure S1).

To assess the impact of relatively heavy bacterial infection on the chemical composition of the blood, concentrations of major electrolytes (sodium, potassium, calcium), pH and oxygen concentration, iron, glucose and lactate were determined at each experimental time point. In addition, complement C3 and total haemolytic complement (CH50) were assayed. Electrolytes, iron and oxygen concentration, and pH, remained in the acceptable range for healthy humans, whether or not bacteria were present (data not shown). Lactate concentration rose, and glucose concentration fell, over the course of the experiments, whether or not bacteria were present. Blood glucose remained in the normal range for fasting humans (3–5.5mmol/L) at all time points except 240 minutes. There was no evidence of complement consumption (as might be reflected by fall in concentrations of C3 and CH50) in the course of the experiment (Table 1).

**Table 1:** Measurements of blood components sampled at time points during blood and *N. meningitidis* co-cultivation.

Blood donor	Addition of bacteria	Bactericidal activity^1^	Time point	Glucose (mmol/l)	Lactate (mmol/l)	C3 (g/l)	CH50 (U/ml)
A	No	n/a	0	4.6	2.7	1.00	nd
A	No	n/a	90	3.7	4.9	0.99	34
A	No	n/a	240	1.9	8.8	1.05	33
A	Yes	Non-killer	90	3.4	4.9	1.00	30
A	Yes	Non-killer	240	1.2	7.9	1.06	26
A+C^2^	Yes	Killer	90	3.6	4.6	1.01	32
A+C^2^	Yes	Killer	240	1.8	8.9	1.07	30
C	No	n/a	0	5.3	1.6	1.08	41
C	No	n/a	90	3.8	4.5	1.08	nd
C	No	n/a	240	2.0	8.7	1.07	nd
C	Yes	Killer	90	3.5	3.9	1.08	42
C	Yes	killer	240	0.8	2.8	1.11	36
Normal range				3.0–5.5	0.5–2.2	0.7–1.7	23–46

1 Bactericidal activities are defined by reduction of CFU, shown in Figure 1A and C.

2 A+C: Blood from donor A with addition of plasma from donor C to 10%.

n/a: not applicable; nd: not determined.

To assess reproducibility of the model, identical experiments were run 4–6 times (biological replicates). The transcriptome of each biological replicate at each time point was compared with that of the first (Rep1) obtained in each group to establish that transcriptional profiles at each time point up to and including 90 min were highly reproducible (correlation >0.91) (Table S1). However, greater variation appeared in transcriptional profiles by 240 min (correlation 0.72–0.94).

### Overall relationship between transcriptomes of each time point groups

The transcriptome of the T = 0min samples (aliquots processed immediately following addition of bacteria to blood) represents early exponential bacterial growth (following 4 hours growth of meningococci freshly restreaked from an overnight culture on to prewarmed fresh sGC plates). To develop a representation of variation over time of the transcriptome of meningococci growing in blood, the transcriptome of Rep1 at the T = 0min time point (Rep1^T = 0^) was compared with all the others. As the period of co-cultivation increased, the correlation fell, indicating a change in the transcriptional profile from as early as 20 min, most striking by 240 min (Table 2). Correlation analysis revealed 58–82% similarity of the Rep1^T = 0^ transcriptome to those of other time points.

**Table 2:** Correlation of transcriptional patterns obtained from biological replicates (T = 0min Rep 1 or Rep1^T = 0^ is the base pattern).

	Sample group name (co-cultivation time points)					
Replicate number	T = 0min	T = 20min	T = 40min	T = 60min	T = 90min	T = 240min
**Rep. 1**	1	0.798	0.823	0.818	0.808	0.708
**Rep. 2**	0.959	0.781	0.803	0.807	0.781	0.689
**Rep. 3**	0.952	0.77	0.796	0.795	0.757	0.585
**Rep. 4**	0.946	0.749	0.795	0.79	0.722	0.575
**Rep. 5**	0.925	0.739	0.795	0.79	0.72	(0.32)
**Rep. 6**	N/A	N/A	0.782	N/A	0.697	N/A

Correlation was calculated using GeneSpring (Agilent) microarray analysis software as described in Material and Methods.

**Figure 2:**
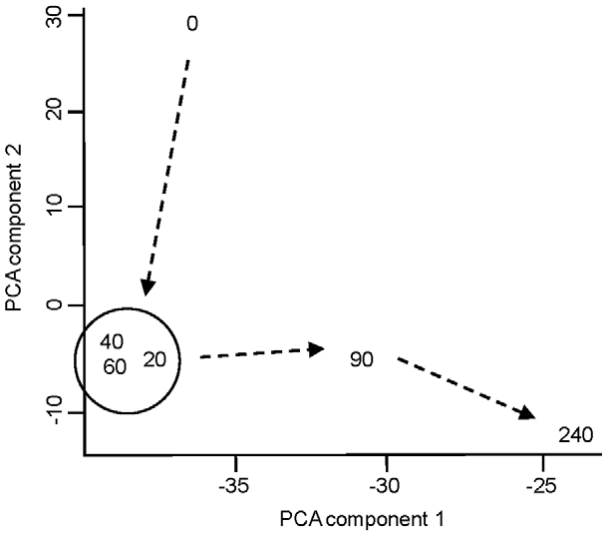
Principal component analysis (PCA) of meningococcal transcriptomes obtained at different blood co-cultivation time points. The numbers in the plot represent the relative positions of samples and co-cultivation time points. Dashed arrows represent the passage of time.

Transcriptome profiles were consolidated for each time point, and variation between profiles at different time points was analysed using Principal Component Analysis (PCA) and displayed in a 2D plot (Figure 2). This showed that the meningococcal transcriptomes at early time points (T = 20min, 40min and 60min) were tightly clustered, suggesting little transcriptional change within this time span. However, major differences between the T = 0min transcriptome and those from T = 20min, 40min and 60min were apparent (as indicated by substantial shift along the PCA component 2 axis). At later time points it appeared that transcription of a different set of genes was altered, indicated by a progressive shift along the PCA component 1 axis.

### Metabolic adaptation and energy conservation

Lists of differentially expressed meningococcal genes were generated by comparing expression data for each co-cultivation time point with that of T = 0min using the same statistical cut-off (1.5-fold change and fault discovery rate p<0.01). In general, the numbers of genes that were up- or down-regulated at each time point were similar, comprising 8–14% of the total genome (2113 ORFs used in array design). The only exception was down-regulated genes for samples at T = 240min which comprised 20% of the total genome (Table S2). When grouped into conventional functional categories, the most differentially expressed genes were energy metabolism, protein synthesis and hypothetical. In the energy metabolism group, many genes encoding enzymes for ATP production were up-regulated. Twelve out of 14 genes of the *nuo* operon encoding subunits for the NADH dehydrogenase complex I [16] were up-regulated at multiple time points (Table S3). The *pet* operon (*petA, petB* and *petC*) encoding the cytochrome c reductase complex III was also up-regulated at multiple time points (Table S3). Four of 8 genes in the *atp* operon encoding ATPase complex V, which produces ATP, were up-regulated at most time points. However, at T = 240min 3 of the 4 genes of the *atp* operon were down-regulated (Table S3).

**Figure 3:**
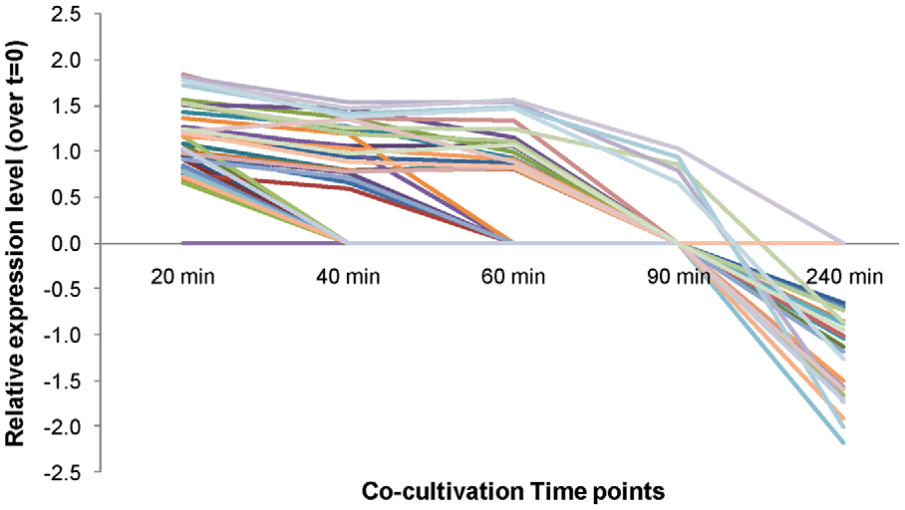
Schematic representation of the number of differentially regulated meningococcal genes. Genes were identified in blood co-cultivation experiments at different time points, which are indicated by colour keys. Genes were classified into TIGR functional categories. Minus signs indicate down-regulated gene number.

**Figure 4:**
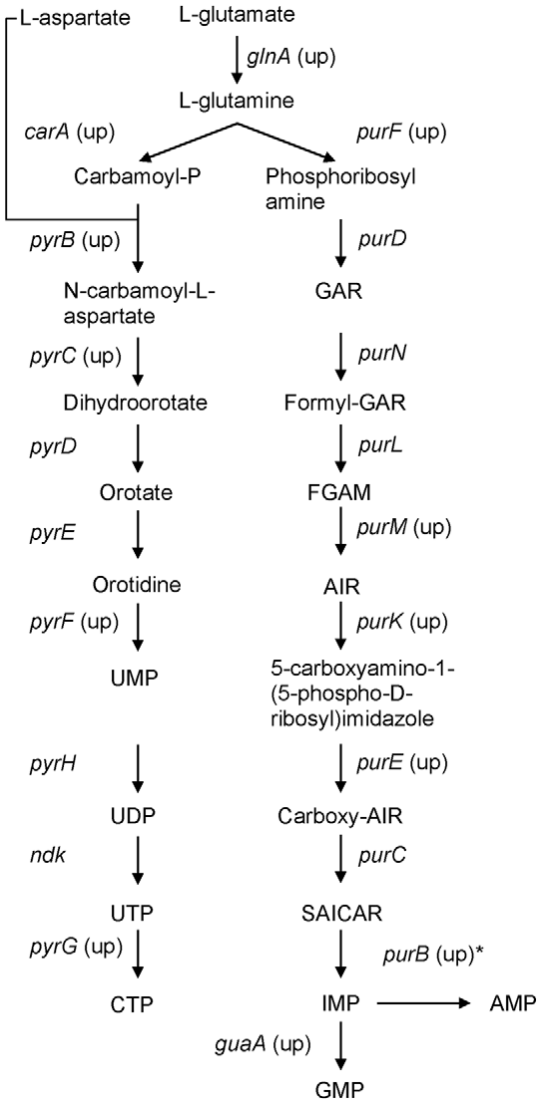
Expression levels of ribosomal protein genes at different co-cultivation time points (relative to T = 0min). Each differently coloured line represents one gene (identified in Table S3).

Most genes in the protein synthesis group were up-regulated at early time points and down-regulated at the T = 240min time point (Figure 3). Further analysis indicated that this difference reflected the differential expression of genes encoding ribosomal proteins. Forty-seven out of 54 ribosomal protein genes were differentially regulated at one or more time points. The trend of differential regulation showed coordinated up-regulation at earlier time points (from T = 20min to T = 90min) and down-regulation at T = 240min (Figure 4, for fold changes see Table S3). Two other groups of genes encoding essential “house-keeping” enzymes/proteins were also down-regulated. These were the DNA gyrase genes *gyrA* and *gyrB*, which were both down-regulated at 40–240min, and cell division genes *minC*, *minD*, *minE*, NMB0191, *ispA*, *ftsZ* and *ftsJ*, which were down-regulated at multiple time points (Table S3). Taken together, these results indicate that meningococci undergo a metabolic adaptation and energy conservation strategy when growing in blood.

As the plasma concentration of lactate increased during the course of co-cultivation, we examined the expression of meningococcal genes involved in its transport and metabolism. The observed change in lactate concentration was not reflected in regulated expression of NMB0543 (*lctP*) encoding lactate permease, nor of NMB1377 (*lldA*) and NMB0901 (both encoding lactate dehydrogenases).

### Meningococcal genes contributing to complement evasion are highly expressed in blood-grown organisms

LOS (lipooligosaccharide), as well as polysaccharide capsule, has been established to be important factors contributing to meningococcal survival in human blood [17]–[19], through distancing activated complement components mediating opsonophagocytosis and complement-mediated killing away from the bacterial surface. The contribution of LOS to serum resistance is importantly enhanced by its sialylation which is catalysed by α-2,3-sialyltransferase encoded by the gene *lst* (NMB0922). This gene was found to be up-regulated at 20, 40, 60 and 240min (Table 3). Other genes encoding enzymes involved in LOS biosynthesis (*lgtA*, *lgtB*, *lgtE*, *lgtF*, and *rfaK*) were also found to be up-regulated at various time points (Table 3). On the other hand, most genes involved in capsular biosynthesis and transport were not found to be significantly differentially up-regulated (Table S3).

**Table 3:** Differentially expressed meningococcal genes encoding virulence factors* during blood co-cultivation compared with T = 0min samples (log_2_ ratio, p<0.01 with FDR).

Gene number	Gene name	Function	Co-cultivation time points				
			20min	40min	60min	90min	240min
NMB0014	*kdtA*	Octulosonic-acid transferase					0.851
NMB0018	*pilE*	Major pilin	0.837	0.714	0.723		
NMB0051	*pilU*	Twitching motility protein	0.618	0.603			−0.602
NMB0179	*fabZ*	3R-hydroxymyristoyl-ACP dehydratase			0.596		0.614
NMB0199	*lpxB*	Lipid-A-disaccharide synthase				0.691	0.657
NMB0216	*katA*	Catalase		2.656	2.750	3.301	2.483
NMB0393		Multidrug resistance protein				0.816	
NMB0586		Putative adhesin		0.649			
NMB0922	*lst*	Alpha-2,3-sialyltransferase	1.159	0.809	0.913		0.686
NMB0926		Opacity protein		0.673			−0.884
NMB0995	*mip*	Macrophage infectivity potentiator	1.337	0.958	0.741	0.734	0.729
NMB1053	*opcA*	Class 5 outer membrane protein	1.124	1.068			
NMB1206	*bfrB*	Bacterioferritin B	2.482	1.193	0.633		1.001
NMB1207	*bfrA*	Bacterioferritin A	2.186	0.969			1.164
NMB1332	*prc*	Carboxy-terminal peptidase					1.217
NMB1704	*lgtF*	Beta-1,4-glucosyltransferase	0.960	0.844	0.884	0.760	0.806
NMB1705	*rfaK*	Acetylglucosamine transferase		0.654			1.041
NMB1779		hemagglutinin/hemolysin					1.057
NMB1880	*fetB2*	Putative ABC transporter, periplasmic solute-binding protein					1.233
NMB1882		TonB-dependent receptor				0.866	
NMB1926	*lgtE*	Lacto-N-neotetraose biosynthesis glycosyl transferase					0.725
NMB1928	*lgtB*	Lacto-N-neotetraose biosynthesis glycosyl transferase					0.600
NMB1929	*lgtA*	Lacto-N-neotetraose biosynthesis glycosyl transferase	0.726		0.904	0.779	0.974
NMB1961		VacJ-related protein	0.742	0.691	0.721	0.634	
NMB2132		Transferrin binding protein-related	0.661				

* as listed by Tettelin *et al*. [6].

*fHbp* (NMB1870) and *nspA* (NMB0663), encoding factor H binding proteins [20], [21], which reduce complement activation at the bacterial surface and so contribute to meningococcal resistance to complement killing, were up-regulated at various time points between 20 and 90 min (Table S3).

### Up-regulation of other known and potential virulence genes promoting survival of meningococci in the human host

Twenty-five out of 104 genes considered by Tettelin *et al*. [6] to encode products contributing to meningococcal virulence were found to be differentially expressed (predominantly up-regulated) at most time points (Table 3). For example, *mip*, encoding macrophage infectivity potentiator (NMB0995), was up-regulated at all time points. The gonococcal homolog is important for *N. gonorrhoeae* survival against macrophage killing [22]. Genes *bfrA* and *bfrB* (encoding bacterioferritin subunits), which are implicated in neisserial iron storage and protection against oxidative stress [23], were up-regulated at most time points. The *katA* gene encoding catalase was highly up-regulated at most time points. The gonococcal homolog was shown to protect *N. gonorrhoeae* from oxidative killing [24].

Genes NMB1962–1966 belonging to the operon encoding the glutamate ABC transporter and accessory machinery [25] were all found to be up-regulated at various time points (Table S3). The operation of this transporter has been implicated in meningococcal resistance to killing in human blood and animal infection models [26] and resistance to neutrophil killing [27], Consistent with previous findings our results further highlight the importance of this operon for survival of meningococci in blood.

Using Gene Set Enrichment Analysis (GSEA) [28], the genes in the amino acid biosynthesis category were found to be significantly enriched for all three time points identified by the programme, 20, 40 and 60 min (Table 4). Further analysis of the category established that most genes in the glutamate families were up-regulated (Table S3), included *gdhA* (NMB1710) and *glnA* (NMB0359). *gdhA*, encoding glutamate dehydrogenase, has been found to be essential for establishing systemic infection in an infant rat model [29].

**Table 4:** Significantly enriched* TIGR functional groups of differentially expressed meningococcal genes in response to blood co-cultivation.

Functional group	Co-cultivation time (min)		
	20	40	60
**Amino acid biosynthesis**	Yes	Yes	Yes
**Biosynthesis, cofactors, prosthetic and carriers**			Yes
**Purines, pyrimidines, nucleosides and nucleotide biosynthesis**	Yes	Yes	Yes
**Protein fate**		Yes	Yes
**Protein synthesis**	Yes	Yes	
**Transcription**	Yes		Yes

* Calculated using GSEA with FDR q-value <0.25.

**Figure 5:**
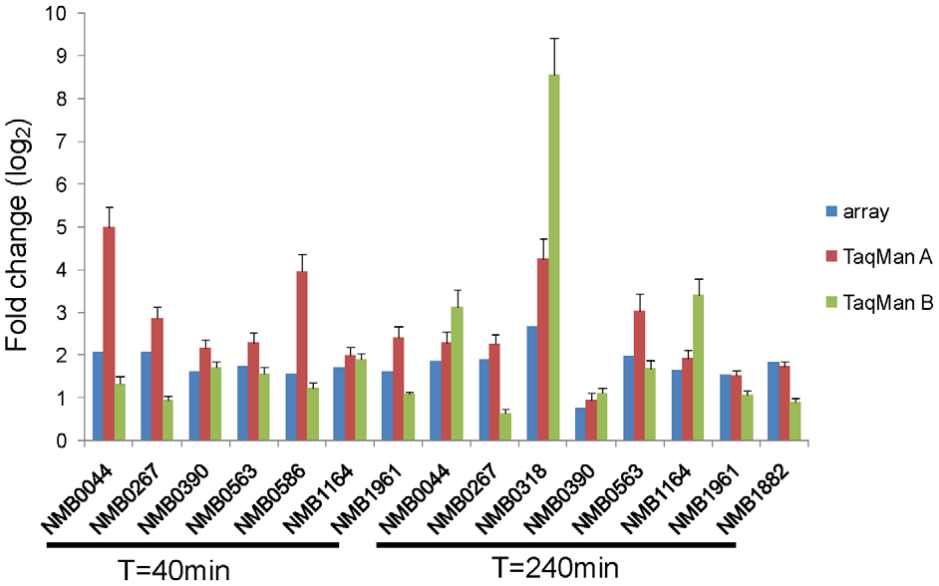
Meningococcal purine and pyrimidine biosynthesis pathway deduced from KEGG pathway database [**49**]. Genes found to be up-regulated are indicated (up). * indicates that PurB catalyzes conversion of SAICAR to IMP and IMP to AMP.

Purine, pyrimidine, nucleoside and nucleotide biosynthesis genes were also identified as highly significantly enriched by GSEA. Most genes encoding enzymes of the purine and pyrimidine biosynthesis pathways were up-regulated at multiple time points (Figure 5 and Table S3). While the contribution of these genes to meningococcal virulence has not been established, genes in the same pathways have been shown to be crucial for survival of *Escherichia coli*, *Salmonella enterica* and *Bacillus anthracis* in human blood [30].

### The stress response is important for meningococcal survival in blood

The up-regulation of genes with roles in promoting meningococcal survival in adverse environments was anticipated to be part of a more generalised stress response induced by bacterial cultivation in blood. Attention was therefore focussed on further genes encoding proteins implicated in the stress response.

The stress response proteins – ClpB, DnaK, DnaJ, GrpE, GroEL and GroES – are proteases or chaperones that work in concert to maintain correct folding of proteins [31]. The cognate genes (*clpB*, *dnaK*, *dnaJ*, *grpE*, *groEL* and *groES*) were found to be up-regulated at most time points (Table 5). Other protease encoding genes that may substitute for *clpB* were found to be up-regulated at multiple time points. These included *lon*, *htpX, clpP* and *clpX,* the last two encoding a two component protease/chaperone complex (Table 5). Components of the NMB1436-NMB1438 operon, which was found to be important for protecting meningococci from oxidative stress and promoting survival in mouse blood model [32], were up-regulated at most time points (Table 5).

**Table 5:** Differentially expressed meningococcal genes encoding stress response proteins during blood co-cultivation compared with T = 0min samples (log_2_ ratio, p<0.01 with FDR).

Gene number	Gene name	Co-cultivation time points				
		20min	40min	60min	90min	240min
**NMB0554**	*dnaK*		1.785	1.507	0.986	
**NMB0561**	*grpE*	0.864	2.397	1.828	1.316	1.564
**NMB0059**	*dnaJ*		1.598	1.180	1.060	
**NMB1972**	*groEL*		2.462	2.260	1.516	1.316
**NMB1973**	*groES*		2.554	2.263	1.435	
**NMB1472**	*clpB*	−0.913	1.145	0.643		
**NMB1231**	*lon*		1.968	1.629	1.006	−0.747
**NMB0822**	*htpX*	1.034	0.991	1.064	0.712	0.734
**NMB1312**	*clpP*	0.961	0.794	0.715		1.472
**NMB1428**	*clpX*	0.782	0.627	0.823		
**NMB1436**		0.866	1.017	1.259	0.642	
**NMB1437**		1.324	1.309	1.626	1.025	0.896
**NMB1438**		1.254	1.344	1.487	1.118	

### Candidate vaccine antigen genes: identification of up-regulated genes encoding surface exposed proteins

Differential gene expression lists were interrogated for genes encoding proteins identified as potential vaccine candidates. Of the three principal antigenic components of the 4CMenB vaccine currently in an advanced stage of development [33] NMB1870 (fHbp) and NMB2132 (NHBA) were up-regulated at 40 and 20 min, respectively, while NMB1994 (encoding NadA) was down-regulated at most time points (Table S3). Genes encoding other potential vaccine candidates such as *nspA* [20], *mip* (NMB0995) [34] and *opcA* [35] were all found to be up-regulated at multiple time points (Table S3).

**Figure 6:**
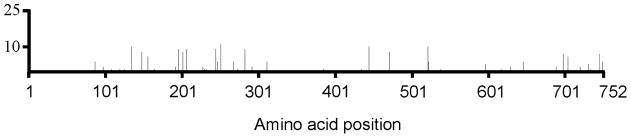
Comparison of gene expression detected by microarray analysis and using real-time RT-PCR (TaqMan). RT-PCR results were obtained using independent blood samples from donor A (TaqMan A) and donor B (TaqMan B) for co-cultivation experiments. Error bars were calculated from three independent biological replicates.

We extended this line of enquiry to identify, for further study, other genes encoding meningococcal proteins known or predicted to be surface exposed which were upregulated during growth in blood and that had not been identified previously as potential vaccine candidates. For nine such genes (Table S3), meningococcal RNA samples obtained from paired co-cultivation experiments in blood from two donors were reverse-transcribed for real-time RT-PCR (TaqMan technology) quantification. Positive correlations were demonstrated between the results of microarray analysis and real-time RT-PCR experiments (Figure 6).

### Surface expression of potential vaccine candidates

**Figure 7:**
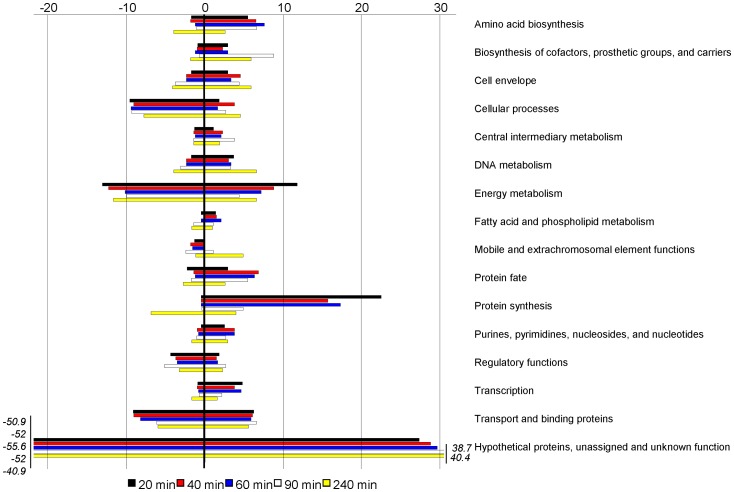
Detection of NMB0390 on the surface of meningococci by FACS. Meningococcal strain MC58 were recovered from human blood (donor A) after 0, 90 and 240 min of cultivation.

Mouse polyclonal antibodies were successfully prepared against the protein products of 4 of these 9 genes (NMB0390, NMB0563, NMB1882 and NMB1961) expressed as recombinants in *E. coli*, and used to assess surface expression experimentally. Increased surface expression of NMB1882, NMB1961 and NMB0563 was not demonstrated, but flow cytometric analysis using anti-NMB0390 showed a progressively increased expression of this protein from T = 90min to T = 240min on the surface of meningococci (Figure 7). Although the cross-reactive signals from the control serum obtained from PBS immunized mice also appeared to be increased with time, the signal from the anti-NMB0390 serum was much greater than that from the anti-PBS serum at later time points: –the signal peaks from anti-NMB0390 and anti-PBS sera were further apart at T = 240min compared to T = 90min. The results indicated that meningococcal surface expression, and therefore potential vaccine candidacy, of NMB0390 was induced by co-incubation with human blood.

**Figure 8:**
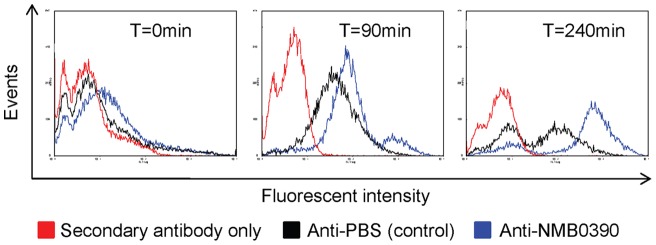
Schematic representation of amino acid variability of NMB0390 homologues from 25 *N. meningitidis* strains. Each vertical bar indicates number of strains carrying different amino acid compared to the consensus sequence of NMB3090 homologues at a position indicated by x-axis. Y-axis indicates number of strains. Strain information is provided in Table 6.

An advantageous characteristic of a good vaccine candidate is a high sequence conservation amongst isolates. Coding sequences for homologues of NMB0390 (maltose phosphorylase) from a total of 25 diverse serogroup B strains were compared. There was a high degree of similarity (≥97%). All translated proteins were 752 amino acids in length, and in all but two positions, just one or other of two possible amino acids were found. Mutations were sparsely scattered throughout the length of the protein (Table 6 and Figure 8).

**Table 6:** Comparison of amino acid sequence between NMB0390 (752 aa) and its homologues from meningococcal isolates.

Isolate Name	Designation^1^	ST^1^	Sequence^3^ identity (%)
**MC58**	B:15: P1.7,16–2	74	100
**BZ169** 2	B: P1.5–2,16	32	100
**H44/76**	B: P1.7,16	32	99
**BZ133** 2	B: P1.7,16	1	98
**M01-240185** 2	B: 2a: P1.5,10–8	11	97
**2996** 2	B: 2b: P1.2	540	97
**BZ198** 2	B: NT: 7–2,4	41	97
**BZ232** 2	B: NT; P1.5–2, 2–2	38	97
**1000** 2	B: P1.ND, ND	20	97
**528** 2	B: P1.5–1,10	18	97
**CU385**	B: 4: P1.15	33	100
**M13399**	B: nd: nd	2976	98
**OX99.30304**	B: nd: nd	44	98
**M0579**	B: NT: P1.5,2	43	97
**961-5945**	B: 2b: P1.21,16	153	97
**ES14902**	B: 2a: P1.5	11	97
**M6190**	B: 2va: P1.5,2	1988	97
**M01-240013**	B: NT: P1.22,9	275	97
**Alpha710**	B: P1.17,16–3: F5–5	136	98
**NZ-05/33**	B: 4: P1.7–2,4	42	98
**M04-240196**	B: NT: P1.19,15	269	98
**M01-240355**	B: 1: P1.22,14	213	98
**M01-240149**	B: 4: P1.7,4	41	98
**G2136**	B: nd: nd	8	97
**961-5945**	B: 2b: P1.21,16	153	97

1 Meningococcal strain information is available at http://pubmlst.org/neisseria.

2 From these meningococcal isolates DNA sequences were determined by sequencing PCR-generated NMB0390 homologues in present study.

3 Amino acid sequence were obtained either from deducing DNA sequences (noted above) or from NCBI and references [50], [51].

## Discussion

Transcriptional profiling offers new insights into mechanisms that allow meningococci to survive in human blood, facilitating formulation of new treatment and prevention strategies.

We have developed a whole human blood experimental system to explore progressive changes in meningococcal gene expression during septicaemia. Biochemical changes developing in the plasma of blood incubated with gentle rocking in a 5% CO_2_–enriched atmosphere even in the absence of meningococci defined a practical limit of 4 hours for experiments, with reduced reproducibility of transcriptomal results after 90 min (Table S1). Replicates of 240 min showed greater variations (0.71–0.94) than the replicates of other time points (91%–98%) (Table S1). Because a stringent p value (0.01) cut off, together with Benjamini and Hochberg error test, was applied to the analysis of samples of all time points, the differences in variation should not impact on the confidence of the genes being selected as differentially expressed. However, 90 min represents ∼3 generations of exponential growth (Figure 1B), long enough for relevant transcriptional changes to occur. Plasma lactate rose consistently in the course of experiments, and while plainly this was not occurring by the same mechanism of tissue ischaemia as seen in septic shock [36], the experimental environment was the more similar to natural meningococcal sepsis as a result.

Microarray technologies offer new opportunities to identify genes or pathways contributing to a complex phenotype such as virulence. Using GSEA [28], we were able to identify a group of genes clustered on purine and pyrimidine nucleotide biosynthesis pathways, which have not been reported previously for their involvement in meningococcal virulence, although identified by others as playing a key role in *E. coli*, *S. enterica* and *B. anthracis* survivial in blood/serum [30]. Glutamine is the substrate for the early stage of *de novo* biosynthesis of purines and pyrimidines. Genes involved in assimilating glutamate from the environment, and converting it to glutamine, were found to be up-regulated during blood co-cultivation. Specific inhibitors of such pathways in bacteria may prove useful as therapeutic agents.

The majority of genes found to be differentially regulated in our study were in pathways relating to amino acid and protein biosynthesis. In particular, ribosomal protein genes showed a switch from up- to down-regulation at early compared to later co-cultivation time points (Figure 3 and 4). This change in regulation coincided with the switch of meningococcal growth from lag to exponential phase (Figure 1B). The change in regulation may, we speculate, reflect metabolic adaptation by N. meningitidis from growth on GC plates to growth in blood.

Echenique-Rivera *et al*. [12] have recently reported results obtained by use of a similar co-cultivation model. They inoculated blood samples with broth-grown meningococci in the early exponential phase of growth. 637 genes were differentially expressed during the course of 15–90 minutes co-cultivation. Comparison of their gene list with ours established that 382 genes were identified as differentially expressed over time in both studies (see Table S3). Genes *fHbp* and *nspA*, *mip* (NMB0995) and *katA*, encoding determinants of bloodstream survival and of virulence, were found to be up-regulated at various time points in both studies, suggesting their importance in this context, while in neither study were genes involved in capsule biosynthesis and transport found to be progressively up-regulated. However, there are many discrepancies between the two studies. For example, the expression of *fur* and other genes involved in the important phenotype of iron uptake (*tbpA*, *tbpB*, *lbpA*, *lbpB*), as well as *lctP*, was found to be up-regulated by Echenique-Rivera *et al*. [12], but down-regulated in our work. We do not consider that such differences invalidate either study, but they do provide an important reminder that results must be interpreted with great caution. Comparing the design of the two studies, there are notable differences. Echenique-Rivera *et al*. [12] used a ten-fold higher starting inoculum (10^8^ CFU/ml blood), a strategy we have found may overwhelm an immune “killer's” ability to contain infection. The bacterial environment in such circumstances must however differ significantly from that encountered by organisms in a non-immune, non-killer’s blood. Echenique-Rivera *et al*. [12] included an RNA amplification step, which is believed to introduce bias to the resulting transcriptomes [15].

Such differences will undoubtedly impact on findings in a major way, but so too will undefined variables such as the nutritional status of the blood donors (whether pre- or post-prandial, affecting levels of lipids, glucose and other energy sources) and their iron status. In short, inferences about host pathogen interaction and the biology of virulence drawn on the basis of changing meningococcal gene expression in the course of such experiments can be no more than tentative, preliminary hypotheses should be further assessed in a better defined setting. These cautionary remarks notwithstanding, the value of pragmatic observations of gene expression should not be underestimated. Surface-exposed antigenic structures which are apparently progressively up-regulated in such experiments remain interesting targets for further study in relation to vaccine discovery.

We identified nine genes apparently falling into this category – their products predicted by the algorithms PSORT-b [37] to have an outer membrane location, and progressively up-regulated during bacterial growth in blood. Depite lacking conventional signal sequences, the product of one of these, NMB0390, appeared in confirmatory FACS experiments with mouse polyclonal serum to be exposed at the meningococcal surface, and so potentially accessible to vaccination-induced bactericidal antibody. Limitations in the quality and quantity of the mouse polyclonal antiserum available have meant that this supportive conclusion as to the surface exposure of NMB0390 cannot at this stage be more than tentative, and further experiments with new reagents, and with an NMB0390 knockout strain, will be needed for confirmation.

In our transcriptome study, NMB0390 was only found to be significantly upregulated at 40 min (Figure 6, Table S3), while a major increase in surface expressed protein was found at 240 min (Figure 7). We suggest that this apparent discrepancy reflects a long half life of the NMB0390 protein compared to its coding mRNA. In prokaryotes (as well as eukaryotes) the abundance of a given mRNA and its coding product is not always correlated [38]. In addition, translational regulation can contribute to the discordance in mRNA – protein abundance [38]. Interestingly, NMB0390 has been predicted to be *down*-regulated in meningococci associated with epithelial cells [39] and also found to be important for meningococcal survival in a mouse infection model [40]. The nasopharynx is the site of (intense) immune selection pressure, and accordingly the exposed domains of meningococcal outer-membrane proteins such as the major porin PorA expressed in this environment are notoriously variable [41], [42]. We have demonstrated in contrast a low level of sequence variation in NMB0390, which we speculate reflects a comparatively low level of expression in the nasopharynx. If its upregulation in the bloodstream is confirmed in further studies, its candidacy for inclusion in a multiple antigen vaccine to prevent serogroup B meningococcal disease [33] would be importantly advanced.

## Materials and Methods

### Bacterial strains and growth conditions

*N. meningitidis* serogroup B strain MC58, used in blood co-cultivation experiments, and 8 others used for NMB0390 sequence comparison, are described in Table 6. Meningococcal strains were routinely propagated on GC (gonococcal) agar (Difco) supplemented with 1% Vitox (sGC) at 37°C in 5% CO_2_ or in Mueller-Hinton broth supplemented with 1% Vitox (Oxoid) at 37°C in an orbital incubator at 180 rpm.

### Human blood co-cultivation model and meningococcal RNA extractions

Human blood was collected from healthy adult volunteers following Imperial College London research guidelines. Specific approval for present study was given by Central Office for Research Ethics Committees, St Mary’s NHS Trust, U.K. under reference number 98/GB/157 (EC3570). Verbal informed consent was obtained from healthy volunteers at the time of the experiments. All volunteers have subsequently given written acknowledgement of their verbal consent and these acknowledgements have been filed along with a Note to File appended to the Investigator Site File and signed by the Investigator, which conforms to the ethics approval.

Human adult blood from donor A or B, both identified as supporting growth of meningococcal strain MC58 in blood (Figure 1B), were used. Due to greater availability, donor A’s blood was used for both microarray and real-time PCR experiments, while donor B’s blood was used only for real-time PCR experiments. For co-cultivation experiments, MC58 was inoculated from an overnight-incubated sGC plate onto fresh sGC plates at a ratio of 10 colonies per 82-mm plate and incubated for 4 hours at 37°C with 5% CO_2_. In each experiment, fresh heparinised blood (used within 20 min of being drawn) was placed in a T25 tissue culture flask (BD Biosciences) and inoculated with 1–5×10^7^ CFU per ml blood of freshly grown MC58 suspended in RPMI1640 (Invitrogen) and the mixture was incubated at 37°C in 5% CO_2_ –enriched air with gentle rocking. In the majority of cases 5–6 ml blood was used for each co-incubation. At desired incubation-time points (0, 20, 40, 60, 90 and 240 minutes) samples (3×100 µl per experiment) were removed for meningococcal CFU enumeration and the remaining co-cultivation contents were mixed with ice-cold 20 ml (4×volume) RPMI1640 containing 5% saponin (Sigma) and incubated on ice for 10 minutes (a procedure which resulted in >95% lysis of human cells, data not shown). Thereafter, 25 ml of RNAprotect bacterial reagent (QIAGEN) was added and incubated at room temperature for >5 minutes. The meningococci were collected by centrifugation at 3,500 g for 10 minutes and the resulting pellets suspended in reagents for RNA extraction using FastRNA Pro Blue kit and FastPrep FP120 (MP Biochemicals) according to the manufactures’ protocols. RNA samples were further treated with DNase (Invitrogen) and purified using an RNeasy kit (QIAGEN).

The quality of meningococcal total RNA and the level of contamination with human total RNA was assessed using the Bioanalyzer 2100 (Agilent) (Figure S1) and the quantity of total RNA was confirmed using a NanoDrop 1000 (Thermo Scientific).

Biochemical tests of blood contents were performed by the Clinical Biochemistry Unit at St Mary's Hospital, Imperial College Healthcare, London, using aliquots of blood samples from co-cultivation experiments.

In addition, plasma from a killer's (donor C) blood was added to blood from donor A to a final 10% in co-cultivation experiments with meningococcal inoculum 10^7^ CFU/ml. At co-cultivation time points 90 min samples were plated out for meningococcal CFU enumeration (Figure 1C). The killer’s blood was also used to characterise bactericidal activity with different meningococcal inoculums (10^9^ and 10^7^ CFU/ml) and meningococcal CFU was obtained at co-cultivation time points 45, 90 and 240 min (Figure 1A).

### DNA microarray hybridization and analysis

Labelling of RNA and DNA samples (5 µg total RNA, 0.5 µg gDNA from strain MC58), microarray hybridization and washing were carried out in accordance with a method previously published [43]. The Pan-Neisseria array slides have been described previously [44]. The array design is available in BµG@Sbase (accession no. E-BUGS-75; http:// bugs.sgul.ac.uk/E-BUGS-75) and also ArrayExpress (accession No. EBUGS-75). Each array slide was co-hybridized with one of the Cy5-labelled experimental RNA samples and Cy3-labelled gDNA (universal reference). The resulting microarray slides were scanned using the GenePix-4000B (Axon) and images analysed with GenePix Pro 6.0 (Axon).

Linear models for microarray data [45] were used to produce differentially expressed gene lists. Background-subtracted spot intensities from hybridized Cy5 labels (signal channel) were divided by the intensities from hybridized Cy3 labels (reference channel). Per slide normalization was performed using median log_2_ ratios. The normalized gene values combined from the biological replicates (n = 5 or 6) of each later time point (20, 40, 60, 90 and 240 min) were compared with that of time zero using linear model implemented in Limma [45] to identify differentially expressed genes with a cut-off value of more than 1.5 fold change (0.585 in log_2_) and p<0.01 adjusted with multiple testing based on Benjamini and Hochberg step down for false discovery rate (FDR) correction [46].

GSEA [28] was performed to identify significantly differentially regulated gene groups according to TIGR functional grouping (www.tigr.org). There was no significant group found in the data for co-cultivation time points 90 min and 240 min.

Correlation of gene expression profiles (slide-to-slide comparison) was calculated using the Find Similar Sample feature in GeneSpring G×7.3 software (Agilent). The gene expression profile of one slide (e.g. T = 0min biological replicate 1) was set as the baseline pattern, and gene expression pattern of any other slide (any replicate of a time point) was compared against that pattern. A similarity score was assigned to each sample based on the degree to which its overall gene expression pattern resembles the baseline pattern.

Fully annotated microarray data have been deposited in BµG@Sbase (accession number E-BUGS-121; http://bugs.sgul.ac.uk/E-BUGS-121) and also ArrayExpress (accession number E-BUGS-121), which is in compliance with MIAME.

### Quantitative real-time RT-PCR

RNA samples from three fresh biological replicates were prepared as described above using blood from donors A and B, and were reverse-transcribed to first strand cDNA using Superscript III and random primers (Invitrogen). Real-time PCR was performed as described previously [43] using TaqMan technologies, TaqMan Universal PCR Master Mix and the ABI 7700 system (Applied Biosystems). The primers were designed and manufactured using Assay-by-Design (Applied Biosystems) and are listed in Table S4. Reporter dye and quencher were FAM and NFQ, respectively. We used 10 ng total RNA per reaction. The reference gene was 16S rRNA. Statistical analysis was based on Livak and Schmittgen [47] and detailed statistical formulations are described in Applied Biosystems’ User Bulletin no. 2 (P/N 4303859).

### Flow cytometry analysis

*N. meningitidis* strain MC58 collected from blood co-cultivation experiments at T = 0, 90 and 240min were suspended in PBS/1%BSA for flow cytometry analysis and diluted to give 10^6^ CFU per reaction, final volume of 40 µl, in wells of 96-well plates. The bacteria were sequentially incubated with firstly antibodies diluted 1∶10 (mouse anti-NMB0390, mouse serum immunised with PBS, or no serum) and secondly antibodies diluted 1∶100 (FITC conjugated anti-mouse serum, from Sigma) at 37°C for 30 minutes with gentle agitation. Bacteria were washed twice with PBS (final volume 190 µl per well) at the end of each incubation and collected by centrifugation at 3,500 g for 10 minutes. After the final wash each bacterial sample were suspended in 150 µl of PBS/0.25% formaldehyde and incubated at 4°C for 16 hours.

The resulting samples were analysed using a flow cytometer CyAn ADP (Dako) with 10^4^ events taken for each sample and Summit v4.3 software (Dako). Meningococcal surface protein specific antibodies together with anti-PBS sera (control sera) were kind gifts from Dr. Barchra Rokbi of Sanofi Pasteur, Lyon, France.

### PCR Generating NMB0390 homologues, sequencing and comparison

Genomic DNA was prepared from a collection of meningococcal strains (Table 6) using QIAamp DNA mini kit (QIAGEN). DNA templates for sequencing were generated by PCR using genomic DNA, HotStar kit (QIAGEN) with 10% DMSO (Sigma) added and primers: TCGGTGTGCGTTTCAATGTG and GTTGTTGTAGTTGTCCAAGTCCAGG at 1 µM each. PCR reactions were performed in an DNA Engine (MJ Research) as 30×(95°C, 30 sec; 52°C, 1 min; 72°C, 3min).

Sequencing products were generated using BigDye v3.1 reaction kit (Applied Biosystems) and primer TGTCGTTTTTACGGCGGGTC, in addition to the two primers used in PCR reaction in order to obtain sequencing on both DNA strands. The products were run on an ABI3730XL sequencer (Applied Biosystems) at Sequencing unit, Department of Zoology, University of Oxford. The resulting sequencing files were checked manually.

The amino acid sequences were compared using ClustalW programme [48]. Protein subcellular localization was predicted using PSORTb programme [37].

## Supporting Information

Figure S1 **Analysis of meningococcal total RNA obtained from co-cultivation experiments using Bioanalyzer 2100.** 16S and 23S represent meningococcal RNA. 18S and 28S represent human RNA.
(TIF)

Table S1 Correlation of transcriptional profiles between individual biological replicates (compared within each biological group against biological replicate 1).
(DOC)

Table S2 Number of differentially regulated meningococal genes during blood co-cultivation.
(DOC)

Table S3 Complete list of differentially expressed meningococcal genes during blood co-cultivation.
(DOC)

Table S4 Primers and probes used for real-time RT-PCR.
(XLS)
