# Correction: Transcriptional Profiling of Serogroup B *Neisseria meningitidis* Growing in Human Blood: An Approach to Vaccine Antigen Discovery

**DOI:** 10.1371/annotation/0727b11a-3671-47a4-a2cf-65098ada6e48

**Published:** 2012-09-28

**Authors:** Asa K. Hedman, Ming-Shi Li, Paul R. Langford, J. Simon Kroll

The links for Tables S3 and S4 were swapped. The link for Table S3 redirects to Table S4 and the link for Table S4 redirects to Table S3. 

