# Correction: Transcriptional Profiling of Serogroup B *Neisseria meningitidis* Growing in Human Blood: An Approach to Vaccine Antigen Discovery

**DOI:** 10.1371/annotation/e273e6f2-2a07-407a-8162-e2547661c192

**Published:** 2012-09-28

**Authors:** Åsa K. Hedman, Ming-Shi Li, Paul R. Langford, J. Simon Kroll

There was a formatting error in the first author's name. Åsa K. Hedman is correct. The correct citation is:

Hedman ÅK, Li M-S, Langford PR, Kroll JS (2012) Transcriptional Profiling of Serogroup B Neisseria meningitidis Growing in Human Blood: An Approach to Vaccine Antigen Discovery. PLoS ONE 7(6): e39718. doi:10.1371/journal.pone.0039718

There were also errors in the placement of Figures 3-8.

The published Figure 7 is actually Figure 3.

The published Figure 3 is actually Figure 4.

The published Figure 4 is actually Figure 5.

The published Figure 5 is actually Figure 6.

The published Figure 8 is actually Figure 7.

The published Figure 6 is actually Figure 8.

