# Supplementary figures and images for: Transcriptional Profiling of Serogroup B Neisseria meningitidis Growing in Human Blood: An Approach to Vaccine Antigen Discovery
Source: PLoS One. 2012 Jun 22;7(6):e39718. doi: 10.1371/journal.pone.0039718 (PMC3382141; doi:10.1371/journal.pone.0039718)

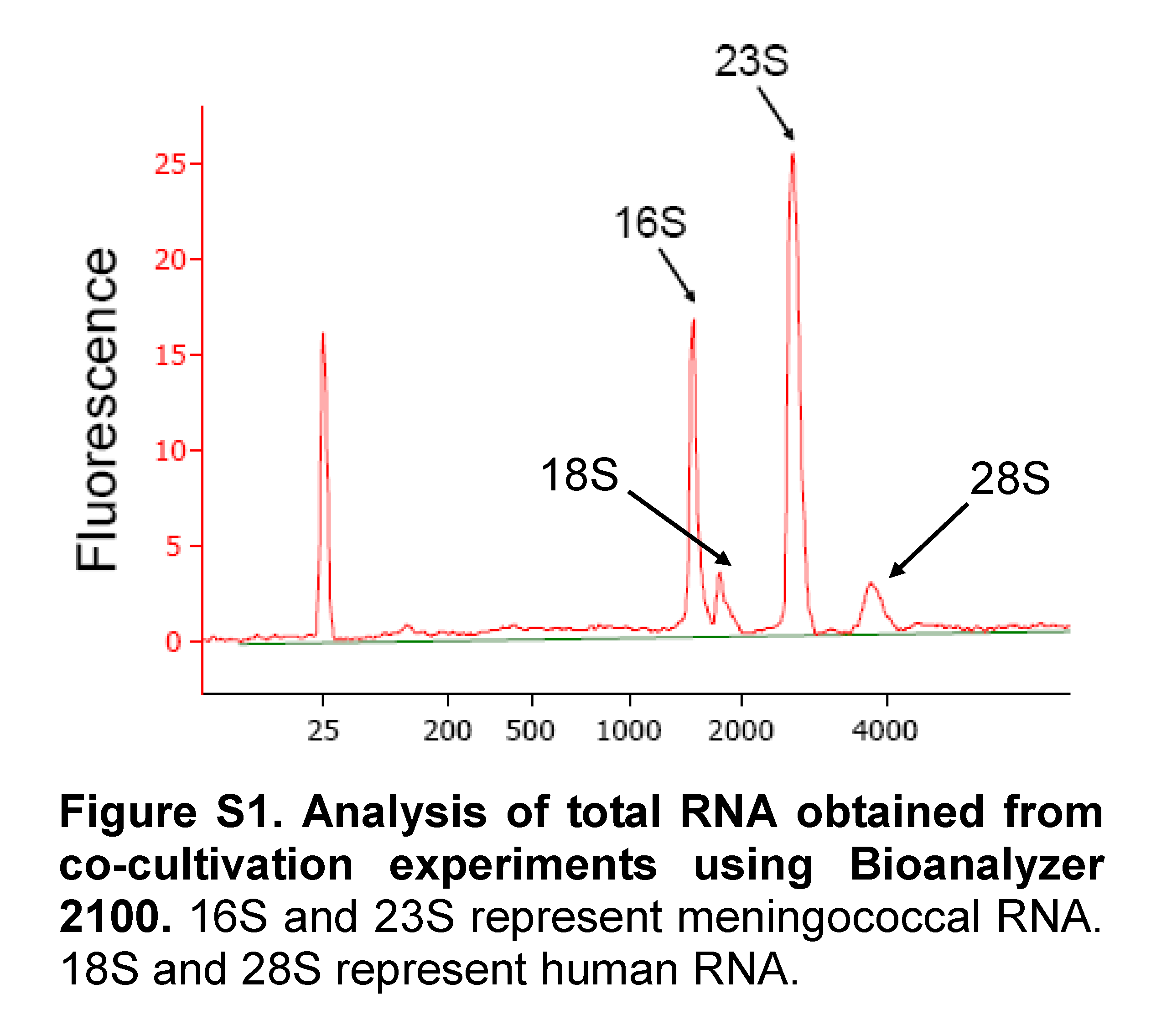

Supplement: Figure S1 — Analysis of meningococcal total RNA obtained from co-cultivation experiments using Bioanalyzer 2100. 16S and 23S represent meningococcal RNA. 18S and 28S represent human RNA. (TIF) [file pone.0039718.s001.tif]
