# Supplementary material for: Transcriptional Profiling of Serogroup B Neisseria meningitidis Growing in Human Blood: An Approach to Vaccine Antigen Discovery
Source: PLoS One. 2012 Jun 22;7(6):e39718. doi: 10.1371/journal.pone.0039718 (PMC3382141; doi:10.1371/journal.pone.0039718)
Supplement: Table S1 — Correlation of transcriptional profiles between individual biological replicates (compared within each biological group against biological replicate 1). (DOC) [file pone.0039718.s002.doc]

Table S1. Correlation of transcriptional profile between individual biological replicates (compared within each biological group against replicate 1)

|  | **Sample group name (co-cultivation time points)** | | | | | |
| --- | --- | --- | --- | --- | --- | --- |
| **Replicate number** | T=0min | T=20min | T=40min | T=60min | T=90min | T=240min |
| **Rep. 1** | 1 | 1 | 1 | 1 | 1 | 1 |
| **Rep. 2** | 0.959 | 0.973 | 0.963 | 0.984 | 0.938 | 0.944 |
| **Rep. 3** | 0.952 | 0.969 | 0.941 | 0.954 | 0.93 | 0.846 |
| **Rep. 4** | 0.946 | 0.962 | 0.924 | 0.942 | 0.924 | 0.83 |
| **Rep. 5** | 0.925 | 0.925 | 0.962 | 0.942 | 0.914 | 0.72 |
| **Rep. 6** | N/A | N/A | 0.951 | N/A | 0.913 | N/A |

*correlation was calculated using GeneSpring (Agilent) microarray analysis software.
