# Supplementary material for: Transcriptional Profiling of Serogroup B Neisseria meningitidis Growing in Human Blood: An Approach to Vaccine Antigen Discovery
Source: PLoS One. 2012 Jun 22;7(6):e39718. doi: 10.1371/journal.pone.0039718 (PMC3382141; doi:10.1371/journal.pone.0039718)
Supplement: Table S2 — Number of differentially regulated meningococal genes during blood co-cultivation. (DOC) [file pone.0039718.s003.doc]

Table S2. Number of differentially regulated meningococal genes during blood co-cultivation

|  | **Co-cultivation time points (min)** | | | | |
| --- | --- | --- | --- | --- | --- |
|  | **20** | **40** | **60** | **90** | **240** |
| Up-regulation | 267 | 252 | 236 | 179 | 303 |
| Down-regulation | 225 | 216 | 257 | 284 | 432 |
