# Supplementary material for: Transcriptional Profiling of Serogroup B Neisseria meningitidis Growing in Human Blood: An Approach to Vaccine Antigen Discovery
Source: PLoS One. 2012 Jun 22;7(6):e39718. doi: 10.1371/journal.pone.0039718 (PMC3382141; doi:10.1371/journal.pone.0039718)
Supplement: Table S3 — Complete list of differentially expressed meningococcal genes during blood co-cultivation. (DOC) [file pone.0039718.s004.doc]

Table S4. Primers and probes used for real-time RT-PCR

| **Gene number** | **Primer type** | **Sequence 5’-3’** |
| --- | --- | --- |
| **NMB0044** | Forward primer | CCACTTTGAAAACTGCGGACAAC |
|  | Reverse primer | CCCAAAATTTAATCAGCGTCGGTTT |
|  | Reporter probe | CCCGCCAGTGTTTACT |
| **NMB0267** | Forward primer | CGCCGCCCACAAAACC |
|  | Reverse primer | CGATGACGCTTTTGCCGTTT |
|  | Reporter probe | ATGTGCGCGTAACCAA |
| **NMB0318** | Forward primer | GTTCAGGTTACGCCGCAAAAG |
|  | Reverse primer | CGTATCGTCGTGCAAAACCTT |
|  | Reporter probe | CCGCACCGTACCGCC |
|  | Reporter probe | CCGTTCAAGCCATATTT |
| **NMB0390** | Forward primer | GCGTTTCAGACAGTCTCTCCTA |
|  | Reverse primer | TCCACGCCGAAGGGATTG |
|  | Reporter probe | CATTGCCGCCCAAACC |
| **NMB0563** | Forward primer | AGCCGGTTCAACCAACACA |
|  | Reverse primer | GCAGTAACGTGTGCGAAGTC |
|  | Reporter probe | CCCTCCGCATTTCAAG |
| **NMB0586** | Forward primer | CCGTCAAACAAAGCAAAGTATCCT |
|  | Reverse primer | GCCTTCTTCTTCGGCTTTGAG |
|  | Reporter probe | AAGCGACCAAAGGCA |
| **NMB1164** | Forward primer | CGGTGTTCGTACCCCGATTT |
|  | Reverse primer | GGAAGCTGGAGCGGTTGT |
|  | Reporter probe | CCGTCGGAACATTCG |
| **NMB1961** | Forward primer | GCTCGATTTGACCGACAGTCT |
|  | Reverse primer | GCGCGTGTAGCTGTATTTGTC |
|  | Reporter probe | ATGGCGGCTTCGTCC |
| **NMB1882** | Forward primer | TGCCGACCATCACCGTTAC |
|  | Reverse primer | CGTGCCGGAAACAGTGTAG |
|  | Reporter probe | CCGTCGTTGGAACTC |
| **16S rRNA** | Forward primer | GCTAATACCGCATACGTCTTGAGA |
|  | Reverse primer | TCGGCCGCTCGAATAGC |
|  | Reporter probe | AAGGTCCCCTGCTTTCT |
